# Metagenomic and metabolomic analysis of gut microbiome’s role in spinal cord injury recovery in rats

**DOI:** 10.17305/bb.2025.12164

**Published:** 2025-03-26

**Authors:** Jieqi Zhang, Xihan Ying, Rong Hu, Yi Huang, Ruoqi Wang, Lei Wu, Dexiong Han, Ruijie Ma, Kelin He

**Affiliations:** 1 Key Laboratory of Acupuncture and Neurology of Zhejiang Province, The Third School of Clinical Medicine (School of Rehabilitation Medicine), Zhejiang Chinese Medical University, Hangzhou, China; 2 Department of Acupuncture, The Third Affiliated Hospital of Zhejiang Chinese Medical University, Hangzhou, China

**Keywords:** Spinal cord injury, SCI, metagenomics, metabolomics, gut microbiome

## Abstract

Spinal cord injury (SCI) induces profound systemic changes, including disruptions in gut microbiome composition and host metabolism. This study aimed to investigate the impact of SCI on gut microbial diversity and serum metabolites in rats, and to explore potential microbiome–metabolite interactions that may influence recovery. Male Sprague–Dawley (SD) rats were assigned to either SCI or sham-operated groups. Fecal samples were collected for whole-genome metagenomic sequencing, and serum samples were analyzed using untargeted metabolomics. Gut microbial composition and diversity were assessed using α- and β-diversity indices, while Linear discriminant analysis effect size (LEfSe) identified differentially abundant taxa. Metabolomic pathway analysis was performed to detect significant changes in serum metabolites, and Spearman’s correlation was used to evaluate associations between gut microbes and metabolites. SCI significantly altered gut microbiota composition, with increased proportions of *Ligilactobacillus* and *Staphylococcus*, and decreased proportions of *Lactobacillus* and *Limosilactobacillus*. Metabolomic analysis revealed disrupted energy metabolism and elevated oxidative stress in SCI rats, as indicated by increased serum levels of pyruvate and lactic acid. Correlation analysis further identified significant associations between specific gut bacteria and key metabolites, suggesting microbiome-driven metabolic dysregulation following SCI. These findings highlight significant interactions between the gut microbiota and host metabolism after SCI and suggest that microbiome-targeted interventions may hold therapeutic potential for improving recovery by modulating metabolic function and oxidative stress responses.

## Introduction

Spinal cord injury (SCI) is a devastating neurological condition that places a significant psychological and economic burden on both patients and their families. Over the past 30 years, the global incidence of SCI has been rising, with an estimated 25,000 to 50,000 new cases occurring annually worldwide [1]. SCI results in the loss of motor, sensory, and autonomic functions, and it also initiates a cascade of complex systemic disorders, including—though not limited to—cardiovascular, respiratory, urinary, and gastrointestinal dysfunctions [2–4]. These widespread effects further complicate rehabilitation and present additional challenges in the treatment of individuals with SCI.

Recent developments in microbiome research have highlighted the significant role of gut microbial communities in human health [5–7]. Loss of central nervous system (CNS) control over the digestive system can lead to marked changes in the gut microbiome [8, 9], which are closely linked to both the recovery process and the onset of systemic diseases following SCI [10]. Moreover, alterations in the gut microbiome can retroactively influence the host, further complicating the biological consequences of SCI [11]. These phenomena are not unique to SCI; similar patterns have been observed in other CNS disorders. For example, studies on patients with traumatic brain injury (TBI) have found that gut microbiome changes are associated with neuroinflammation, cognitive decline, and behavioral issues [12]. Likewise, research on neurodegenerative diseases such as Alzheimer’s disease suggests that microbial imbalances may contribute to disease progression [13]. Together, these findings underscore a complex and dynamic interplay between the gut microbiome and the CNS, indicating that targeting this interaction could present novel strategies for treatment or symptom alleviation.

In this study, we utilized SCI rat models to perform a comprehensive analysis of stool and serum samples from both 14-day post-injury SCI rats and sham-operated controls. We employed fecal metagenomic shotgun sequencing and non-targeted serum metabolomics to assess structural and functional changes in the gut microbiome following SCI, and to explore potential associations between these changes and the host’s metabolic state. We hypothesized that, compared to the sham group, SCI model rats would exhibit significant alterations in gut microbiome composition, which may be closely linked to shifts in host metabolism. Through this research, we aim to deepen our understanding of the complex interactions underlying the effects of SCI and to provide new insights into common gut microbiome changes associated with CNS diseases in humans, such as TBI and Alzheimer’s disease.

## Materials and methods

### Animals

Due to the higher prevalence of SCI in male patients, male Sprague–Dawley (SD) rats were used in this study. Healthy adult male SD rats (8 weeks old, weighing 200–220 g) were obtained from Shanghai Xipu Bikai Experimental Animal Company (animal license No. SCXK (Shanghai) 2018-0006) and housed at the Laboratory Animal Center of Zhejiang Chinese Medical University, which is accredited by the Association for Assessment and Accreditation of Laboratory Animal Care (AAALAC; animal license No. SYXK (Zhejiang) 2018-0012). Rats were maintained under controlled conditions with free access to food and water. To ensure equal access, food and water baskets were placed at a lower height in the cages, with supplies provided in sufficient quantities and monitored daily. At the end of the experiment, all animals were euthanized via overdose of anesthesia in accordance with ethical guidelines.

In this study, only male rats were used to minimize variability due to hormonal fluctuations, as estrogen levels in female rats can significantly influence gut microbiome composition and metabolic profiles. Sixteen rats were randomly assigned to either the SCI group (*n* ═ 8) or the sham group (*n* ═ 8), with four rats housed per cage. All animal experiments were conducted in accordance with the protocol approved by the Animal Ethics Committee of Zhejiang Chinese Medical University (IACUC-20230313-01) and strictly followed the National Institutes of Health (NIH) guidelines for the care and use of laboratory animals (NIH Publication No. 8023).

### SCI model

Rats were anesthetized via intraperitoneal injection of sodium pentobarbital (30 mg/kg) and placed on a constant-temperature surgical table for the procedure. Following surgical exposure of the T10 spinal cord segment, a moderate SCI was induced using the NYU impactor system, which delivered a computer-controlled impact with a 10-g weight dropped from a height of 5 cm, targeting the T10 segment. The skin was sutured after the surgical site was cleaned. Indicators of successful model establishment included spasmodic twitching, tail flicking, dural congestion, or hematoma. Rats that regained consciousness with a Basso, Beattie, and Bresnahan (BBB) locomotor score of 0–2 were considered to have been successfully modeled [14]. In the sham group, the vertebral plate was removed to expose the spinal cord, but no impact was applied. Postoperative care for all animals, including both the sham and SCI groups, involved intraperitoneal injections of penicillin (100 U/day) for the first three days. Additionally, rats in the model group received twice-daily abdominal massages to assist with urination until they were able to urinate independently.

### Sample collection and preparation

Fourteen days after the SCI model was established, each rat was placed in a separate sterile cage. At least two fecal pellets were collected from each rat, placed in sterile conical tubes, rapidly frozen in liquid nitrogen, and stored at –80 ^∘^C for subsequent microbiome analysis. At the end of the experiment, blood was collected under anesthesia with sodium pentobarbital via the abdominal aorta. Serum was then isolated and stored at –80 ^∘^C.

### Metagenome DNA extraction and shotgun sequencing

Microbial DNA from all samples was isolated using the OMEGA Mag-Bind Soil DNA Kit (M5635-02) (Omega Bio-Tek, Norcross, GA, USA), following the manufacturer’s guidelines, and stored at –20 ^∘^C for future analysis. The concentration and purity of the isolated DNA were assessed using a Qubit™ 4 Fluorometer with WiFi (Q33238), Qubit™ Assay Tubes (Q32856), and the Qubit™ 1X dsDNA HS Assay Kit (Q33231) (Invitrogen, USA), as well as agarose gel electrophoresis. The extracted microbial DNA was then used to construct metagenomic shotgun sequencing libraries with 400 bp insert sizes using the Illumina TruSeq Nano DNA LT Library Preparation Kit. Metabo-Profile Biotechnology Co., Ltd. (Shanghai, China) performed sequencing on all libraries using the Illumina NovaSeq platform (Illumina, USA) with the PE150 strategy.

The complete microbial genomic DNA was isolated using the OMEGA Mag-Bind Soil DNA Kit (M5635-02) from Omega Bio-Tek (Norcross, GA, USA), following the manufacturer’s guidelines. The extracted DNA was then stored at –20 ^∘^C for future analysis. DNA quantity and purity were assessed using the Qubit™ 4 Fluorometer with WiFi (Q33238), along with Qubit™ Assay Tubes (Q32856) and the Qubit™ 1X dsDNA HS Assay Kit (Q33231) from Invitrogen, USA. Agarose gel electrophoresis was also performed for quality assessment. The Illumina TruSeq Nano DNA LT Library Preparation Kit was used to process the isolated microbial DNA and construct metagenomic shotgun sequencing libraries with an insert size of 400 bp. Sequencing was carried out using the Illumina NovaSeq platform (Illumina, USA) with a PE150 strategy by Metabolo-Profile Biotechnology Co., Ltd. (Shanghai, China).

### Metagenomics analysis

Raw sequencing reads were quality-filtered and taxonomically classified using Kraken2 against a RefSeq-derived database. Samples were then assembled with MEGAHIT, and contigs longer than 300 bp were clustered using MMseqs2. To retain only relevant contigs, sequences were aligned against the NCBI-nt database to identify and exclude non-target taxa. Gene prediction was performed using MetaGeneMark, and gene abundance was quantified by mapping reads to predicted sequences with Salmon, followed by normalization to counts per million (CPM). Functional annotation of genes was conducted against the KEGG, EggNOG, and CAZy databases using MMseqs2. Additional annotations were generated using EggNOG-mapper and KOBAS to provide enriched biological insights.

### Serum extraction and metabolomics analysis

Chemicals for targeted metabolites were obtained from Sigma-Aldrich, Steraloids Inc., and TRC Chemicals, and were dissolved in appropriate solvents to prepare stock solutions. For sample preparation, plasma samples were thawed on ice, added to wells, and processed with methanol and internal standards using an Eppendorf epMotion Workstation. This was followed by centrifugation and derivatization. Analytical measurements were performed using an ACQUITY UPLC-Xevo TQ-S system (Waters Corp.) equipped with a UPLC BEH C18 column (1.7 µm, 2.1 × 100 mm) and a VanGuard precolumn (1.7 µm, 2.1 × 5 mm). The mobile phase consisted of: A) water with 0.1% formic acid, and B) a 70:30 mixture of acetonitrile and isopropanol. The gradient conditions were: 0–1 min (5% B), 1–11 min (5%–78% B), 11–13.5 min (78%–95% B), 13.5–14 min (95%–100% B), 14–16 min (100% B), 16–16.1 min (100%–5% B), and 16.1–18 min (5% B). The flow rate was set to 0.40 mL/min, and the column temperature was maintained at 40 ^∘^C. The injection volume was 5 µL. Quality control measures included the use of internal standards and pooled QC samples to ensure data consistency and reliability. UPLC-MS/MS data were processed using TMBQ software for peak integration and quantification, followed by statistical analysis to interpret the metabolic profiles.

### Ethical statement

Ethical approval was obtained from the Animal Ethics Committee of Zhejiang Chinese Medical University (I ACUC-20230313-01).

### Statistical analysis

Statistical analyses and data visualization were performed using R software (version 4.1.2). Descriptive statistics—including means and standard deviations for continuous variables, and frequencies and percentages for categorical variables—were calculated. Group comparisons were conducted using *t*-tests for continuous variables and chi-squared tests for categorical variables. β-diversity was assessed using Bray–Curtis distances and visualized via principal coordinate analysis (PCoA). MaAsLin2 was used to identify differentially enriched taxa and their associated functions. Metabolomic data were analyzed using orthogonal partial least squares discriminant analysis (OPLS-DA) and univariate tests (*t*-test and Mann–Whitney *U* test). Multiple testing corrections were applied as appropriate. Statistical significance was defined as *P* < 0.05.

## Results

### SCI induces significant changes in gut microbial composition and diversity

**Figure 1.**
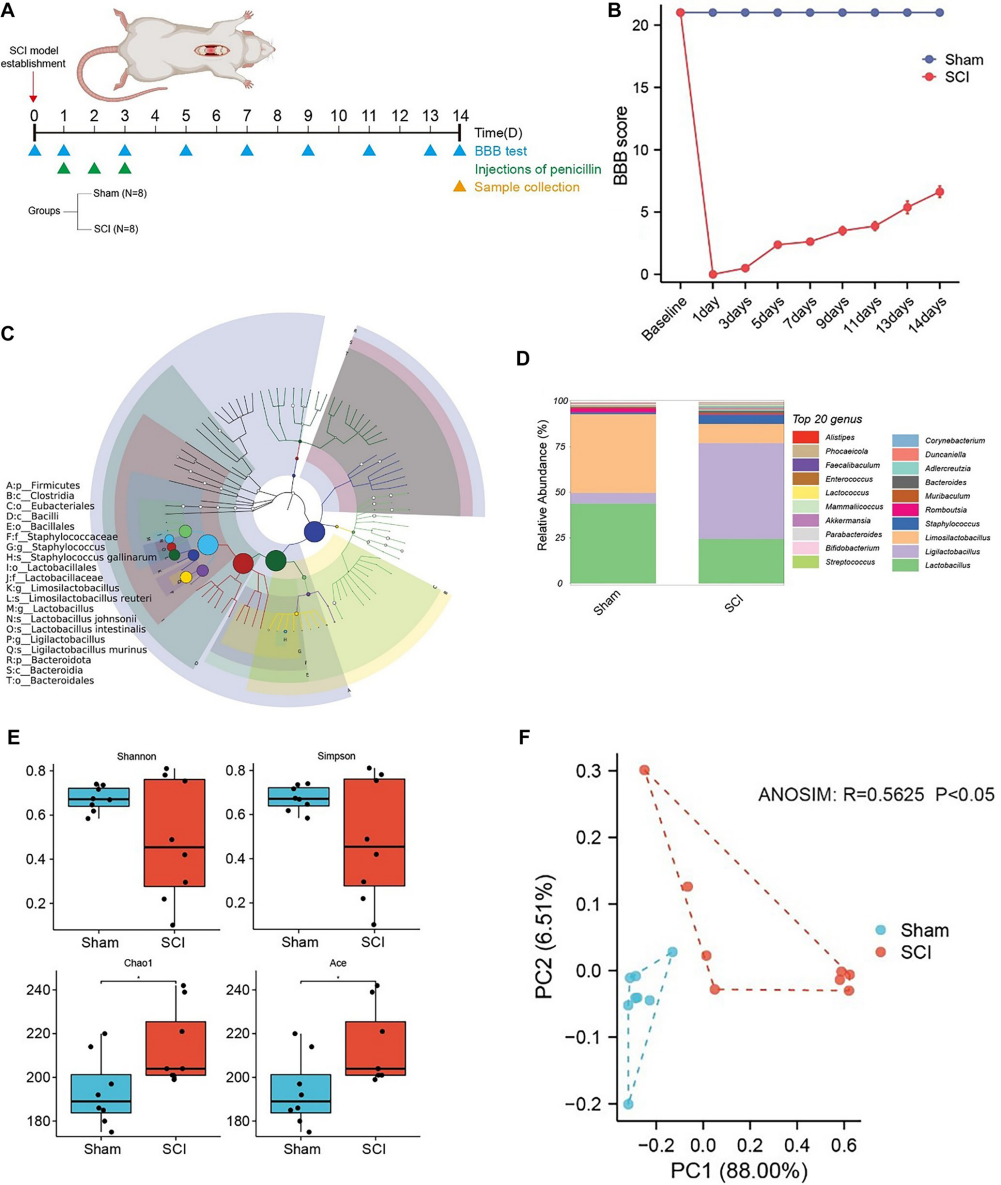
**Impact of SCI on gut microbial composition and diversity in rats.** (A) Schematic diagram of the experimental design, detailing the sample size and group allocation, the timeline of SCI modeling, penicillin injection, BBB testing, and sample collection; (B) Line chart showing the BBB scores over time; (C) Cladogram illustrating overall species composition, diversity, and abundance distribution. In this figure, the classification rank tree from the inner circle to the outer circle shows the rank relationship of all taxa (represented by nodes) from phyla to species in the sample population, and the node size corresponds to the average relative abundance of the taxa. The top 20 taxa in the relative abundance are also identified by letters in the figure (from phyla to genus in order from outer layer to inner layer). The shadow on the letter is the same color as the corresponding node; (D) Stack bar plots of the top 20 dominant species at the genus level between SCI and sham groups; (E) Boxplots of species α-diversity between the SCI group and sham group. Mann–Whitney *U* test, *P < 0.05; (F) PCoA plots showing clear clustering separation between SCI and sham groups. ANOSIM: R ═ 0.5625, *P* < 0.05. SCI: Spinal cord injury; BBB: Basso, Beattie, and Bresnahan; PCoA: Principal coordinate analysis.

To investigate changes in intestinal microbiome composition and diversity in SCI rats, this study employed whole-genome shotgun sequencing of fecal samples to compare the gut microbiome between the sham and SCI groups. The experimental timeline outlines key procedures, including the establishment of the SCI model, BBB testing, and sample collection (Figure 1A). Recovery was assessed over 14 days using the BBB test. On the first day after surgery, all rats in the SCI group had a BBB score of 0, which gradually increased over time. In contrast, the sham group maintained a consistent BBB score of 21 throughout the study (Figure 1B). A cladogram was constructed to illustrate the overall species composition, diversity, and abundance distribution (Figure 1C). At the genus level, stacked column charts of the top 20 dominant species revealed an increased proportion of *Ligilactobacillus* and *Staphylococcus* in the SCI group, whereas the proportions of *Lactobacillus* and *Limosilactobacillus* decreased (Figure 1D). α-Diversity analysis showed significant increases in the Chao1 and ACE indices in the SCI group, suggesting that SCI may promote the richness of rare or previously undetected species (Figure 1E). β-Diversity analysis using PCoA plots demonstrated clear clustering separation between the SCI and sham groups. ANOSIM analysis yielded an *R* value of 0.5625 and a *P* value < 0.05, confirming significant differences in microbial composition between the two groups (Figure 1F).

### Key differences in gut microbial community structure following SCI

**Figure 2.**
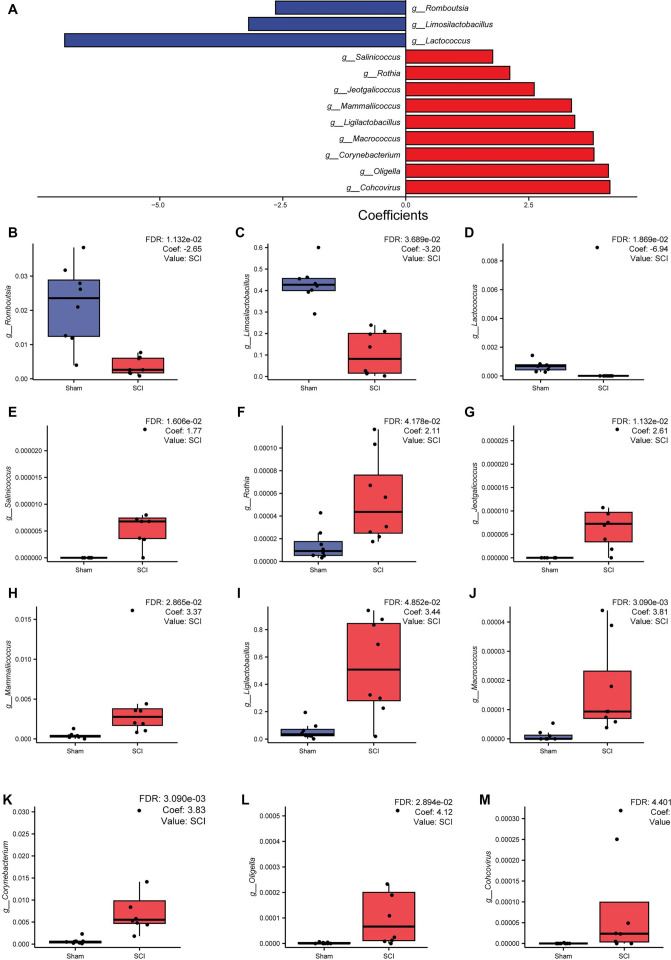
**Key species differences of gut microbiome after SCI.** (A) The bidirectional bar plot shows the results of the MaAsLin2 analysis (FDR < 0.05). The left side represents genera with decreased coefficients in the SCI group compared to the Sham group, while the right side represents genera with increased coefficients in the SCI group compared to the sham group. Blue bars indicate a decrease, red bars indicate an increase, and the length of the bars reflects the magnitude of the coefficient values. (B–M) Box plots illustrating the relative abundance of key genera identified through MaAsLin2 analysis, comparing the SCI group to the sham group. The coefficients (coef) and FDR values are indicated in the top-right corner of each plot. SCI: Spinal cord injury; FDR: False discovery rate.

Further MaAsLin2 analysis revealed significant differences in key genera between the SCI and sham groups, with the coefficients indicating changes in the relative abundance of microbiota in the SCI group compared to the sham group. A bidirectional bar chart was generated to illustrate the coefficients (coef) of key genera showing significant changes in the SCI group (Figure 2A).

These differences in microbial composition were further illustrated using box plots (Figure 2B–2M), which confirmed significant shifts between groups. Specifically, *Corynebacterium*, *Macrococcus*, *Mammaliicoccus*, *Oligella*, and *Jeotgalicoccus* were more abundant in the SCI group. In contrast, genera, such as *Lactococcus*, *Limosilactobacillus*, *Romboutsia*, and Lactobacillus were significantly reduced compared to the sham group. These findings suggest that SCI induces a shift in gut microbiome composition, marked by an increase in potentially immune-modulating or proinflammatory genera and a decrease in those typically associated with gut health and barrier function.

### Impact of SCI on functional features of gut microbiome

**Figure 3.**
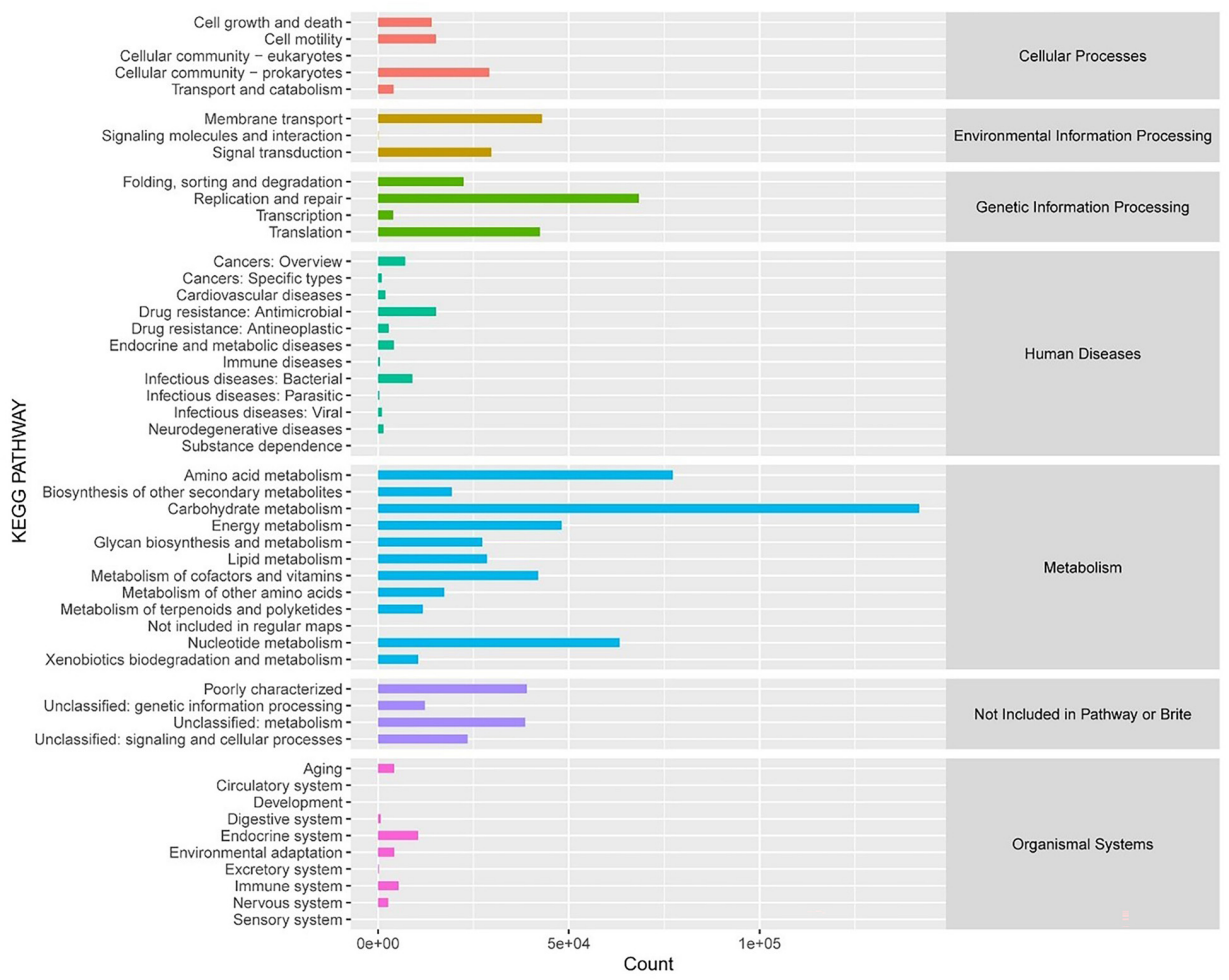
**Statistical map of KEGG metabolic pathway annotation results.** In the figure, the horizontal coordinate is the number of proteins annotated to the corresponding metabolic pathway, and the vertical coordinate corresponds to each metabolic pathway of KEGG’s second grade, and the classification of the first grade to which each metabolic pathway belongs is listed on the right.

**Figure 4.**
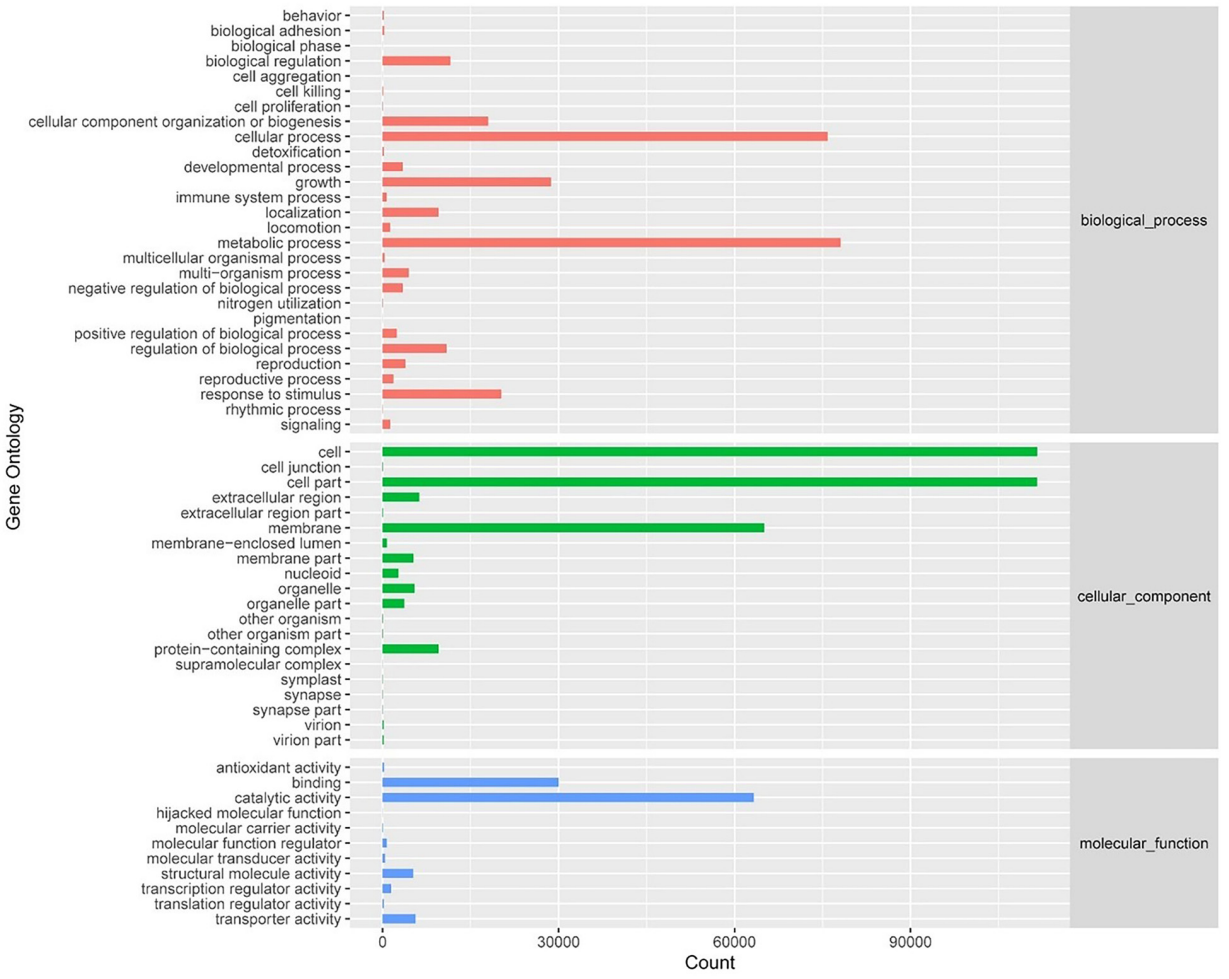
Statistical map of GO Slim annotation results.

**Figure 5.**
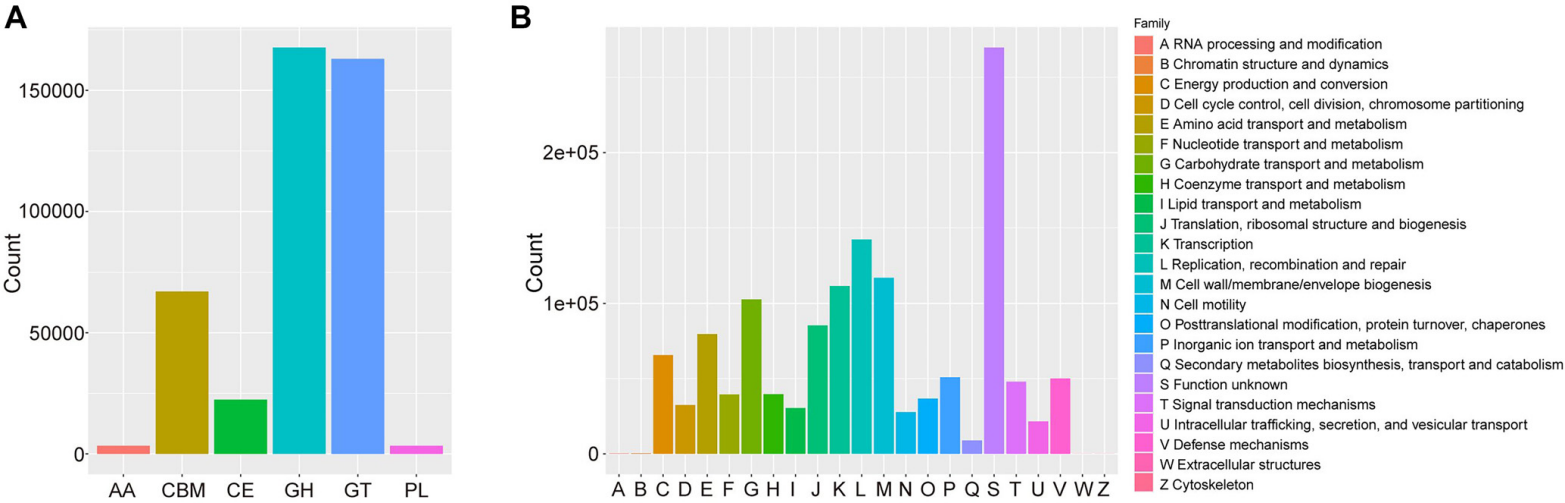
**CAZy and EggNOG annotation results.** (A) Statistical diagram of annotation results of CAZy enzyme function module. In the figure, the horizontal coordinate corresponds to each CAZy enzyme function module, and the vertical coordinate is the number of protein families annotated to the corresponding module; (B) EggNOG function group annotation statistics. In the figure, the horizontal coordinate corresponds to the 25 functional categories of eggNOG genes, each of which is represented by an English capital letter. AA: Auxiliary activities; CBM: Carbohydrate-binding modules; CE: Carbohydrate esterases; GH: Glycoside hydrolases; GT: Glycosyl transferases; PL: Polysaccharide lyases.

Using databases, such as KEGG metabolic pathways, eggNOG functional categories, CAZy enzyme families, and GO functional groups, we evaluated the functional characteristics of the gut microbiome in rats with sham surgery and SCI. KEGG pathway analysis revealed significant enrichment in key metabolic pathways, including carbohydrate, amino acid, lipid, and energy metabolism (Figure 3). GO analysis showed enrichment in the metabolic process category under biological processes (BPs) and antioxidant activity under molecular functions (MFs) (Figure 4). CAZy analysis indicated the enrichment of Glycoside Hydrolases (GHs), Glycosyl Transferases (GTs), and Carbohydrate-Binding Modules (CBMs) among the gut microbiome (Figure 5A). EggNOG analysis further highlighted the microbiome’s potential impact on carbohydrate metabolism (Figure 5B). These findings suggest that the gut microbiome may play roles in host metabolism and the oxidative stress response following SCI.

### Functional differences in gut microbiome following SCI

**Figure 6.**
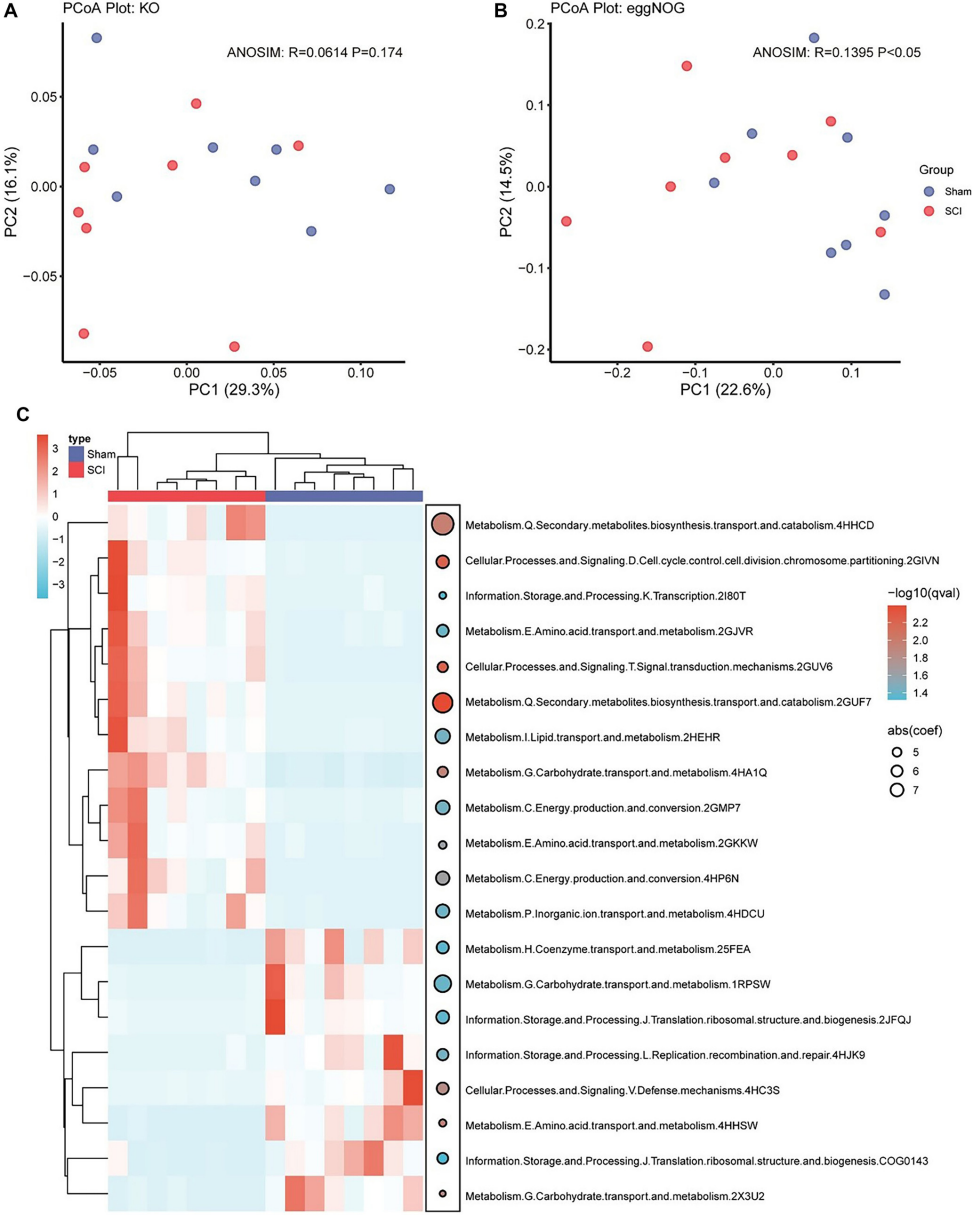
**Key differences in gut microbial community structure after SCI.** (A) PCoA plot of functional enrichment results based on the KO database. ANOSIM: R ═ 0.0614, *P* ═ 0.174; (B) PCoA plot of functional enrichment results based on the eggNOG database. ANOSIM: R ═ 0.1395, *P* < 0.05; (C) Heatmap illustrating the coefficients and FDR of enriched pathways identified through MaAsLin2 analysis. On the right, bubble size represents the absolute value of the coefficients, with larger bubbles indicating larger absolute values. The color of the bubbles reflects the FDR values (FDR < 0.05). SCI: Spinal cord injury; PCoA: Principal coordinate analysis; KO: KEGG Orthology; FDR: False discovery rate.

Next, we compared the functional profiles of the gut microbiota between the SCI and sham groups to assess the impact of SCI on intestinal microbial function. We first conducted PCoA and ANOSIM. The KEGG Orthology (KO) analysis did not reveal significant functional differences between groups (*R* ═ 0.0614, *P* ═ 0.174) (Figure 6A). However, the eggNOG analysis indicated a slight but statistically significant difference (*R* ═ 0.1395, *P* < 0.05), suggesting weak intergroup functional variation (Figure 6B). To further explore these differences, we used the MaAsLin2 package. Heatmaps and dot plots were generated to visualize the top 20 most significant pathways (ranked by coefficient values). Only pathways with a false discovery rate (FDR) < 0.05, excluding those classified as “unknown,” were considered (Figure 6C). Within the “Metabolism” category, several significantly enriched pathways were identified, including secondary metabolite biosynthesis, lipid transport, and energy production and conversion. These findings suggest that SCI-induced alterations in the gut microbiota may impact metabolic processes—particularly those related to energy metabolism and lipid biosynthesis—which could be critical for understanding the pathophysiological consequences of SCI.

### Significant changes in host serum metabolites due to SCI

**Figure 7.**
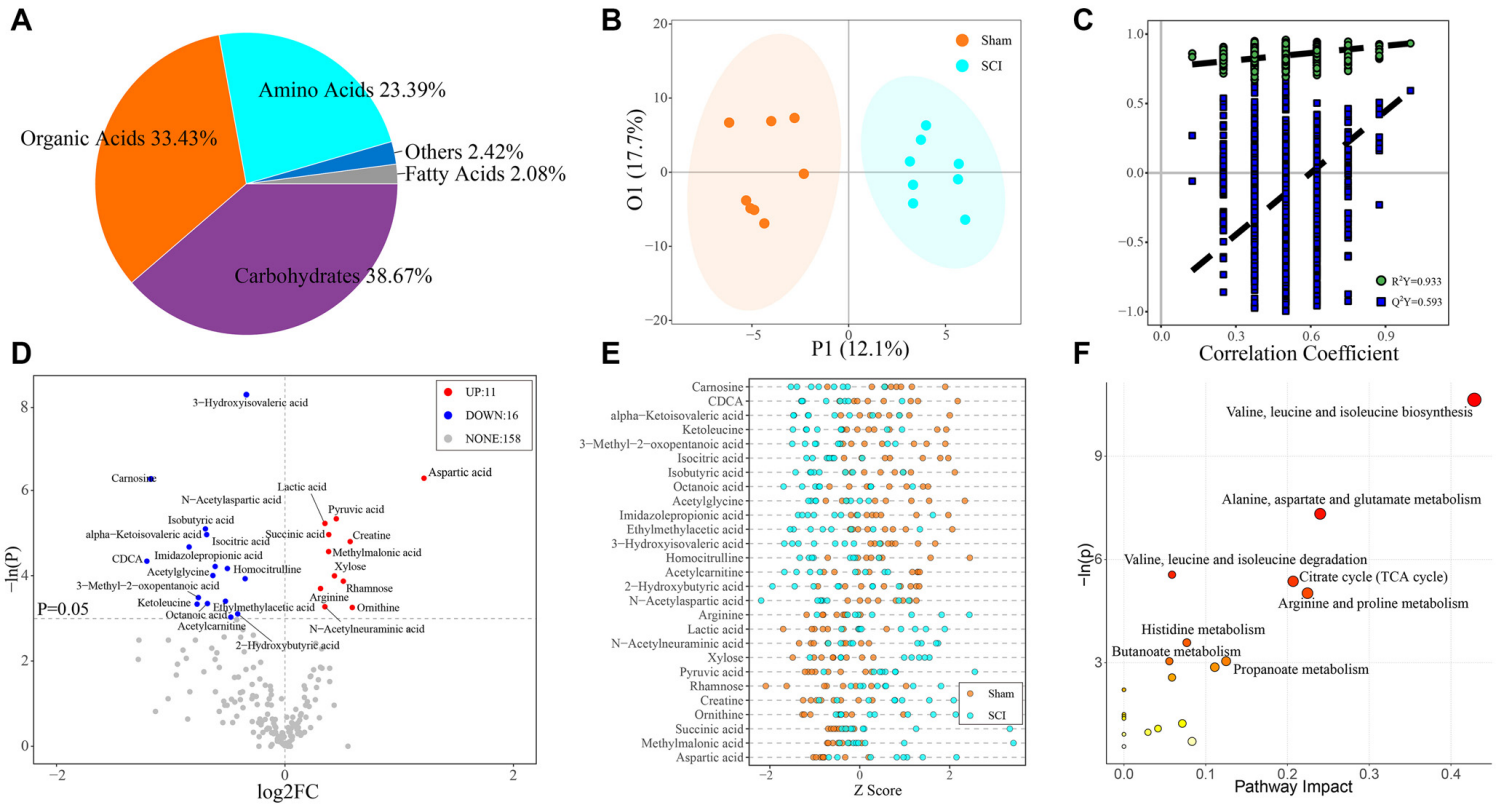
**Significant changes in host serum metabolites due to SCI.** (A) Pie plot of the proportion of identified metabolite classes in all samples; (B) OPLS-DA score plot; (C) Permutation plot of OPLS-DA; (D) Volcano plot of univariate statistics. |log2FC| > ═ 0, *P* < 0.05. All metabolites were derived from VIP > 1 metabolites in OPLS-DA; (E) Z Score dot plot of the differential metabolites; (F) Pathway analysis bubble plot by rno set. *P* < 0.05. SCI: Spinal cord injury; OPLS-DA: Orthogonal partial least squares discriminant analysis.

To investigate the interaction between changes in gut microbiome composition and host metabolism, we conducted an untargeted metabolomic analysis of host serum. A pie chart (Figure 7A) illustrates the distribution of all detected serum metabolites by category, highlighting the relative proportions of the main components. The results showed that carbohydrates (38.67%) and organic acids (33.43%) were the predominant metabolites, followed by amino acids (23.39%). Fatty acids (2.08%) and other categories (2.42%) were present in smaller amounts.

OPLS-DA further revealed clear differences in metabolic features between the two groups, as shown in the score plots (Figure 7B) and validated by the model permutation test (Figure 7C). Based on the OPLS-DA model, we identified key metabolites contributing to group differences by assessing their importance to the model (VIP, Variable Importance in Projection) and their reliability (correlation coefficients with the first principal component). Metabolites with VIP > 1 were considered significantly different between groups.

Further univariate testing identified 27 metabolites that differed significantly between the two groups. These are visualized in a volcano plot (Figure 7D), generated exclusively from the metabolite set with a VIP > 1 based on OPLS-DA. A corresponding Z-score plot (Figure 7E) displays the standardized expression differences of these metabolites, offering a clear visual comparison.

The analysis results indicated that elevated levels of pyruvic acid and lactic acid in the SCI group suggest disordered energy metabolism and local tissue hypoxia. A decrease in carnosine levels reflects increased oxidative stress in SCI patients. Higher aspartic acid levels may be associated with altered neurotransmitter activity. Meanwhile, reduced levels of 3-hydroxyisovaleric acid point to changes in fatty acid metabolism pathways, potentially linked to energy production and inflammatory responses.

We also conducted a pathway analysis of these 27 metabolites using the rno library (Figure 7F), identifying significant enrichment in eight pathways (*P* < 0.05). These pathways are primarily associated with energy metabolism, amino acid metabolism, and processes related to inflammation and nerve repair. Key pathways include the citrate (TCA) cycle; the biosynthesis and degradation of branched-chain amino acids, such as valine, leucine, and isoleucine; the metabolism of alanine, aspartate, and glutamate; and the metabolism of arginine and proline. Additional important pathways include histidine metabolism, as well as butyrate and propionate metabolism.

In summary, the metabolic changes induced by SCI include disrupted energy metabolism, increased oxidative stress, altered neurotransmitter activity, and changes in metabolic pathways. These findings offer valuable insights into the biological basis of SCI and help guide the development of effective treatment strategies.

### Potential links between gut microbiome and serum metabolites in SCI

**Figure 8.**
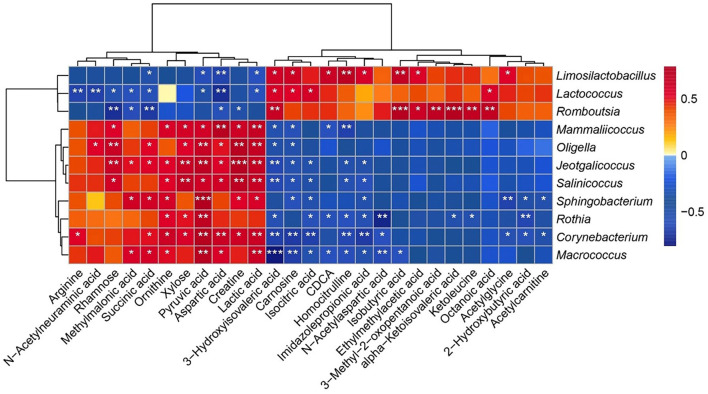
Spearman correlation heatmap between gut microbes and serum metabolites (*R* > 0.7, *P < 0.05, **P < 0.01, *** *P* < 0.001).

Finally, we employed Spearman’s correlation analysis to explore the relationships between the gut microbiome and serum metabolites, with the results presented in a heatmap (Figure 8). Notably, *Limosilactobacillus* showed a positive correlation with carnosine and 3-hydroxyisovaleric acid, both of which are involved in amino acid synthesis and degradation. *Lactococcus* was positively correlated with isocitric acid, a key intermediate in energy metabolism, while *Romboutsia* showed positive correlations with isobutyric acid and ethylmethylacetic acid, both related to short-chain fatty acid (SCFA) metabolism. These findings highlight the complex interactions between the gut microbiome and host metabolites and offer new insights into the mechanisms by which SCI affects host physiology.

## Discussion

Our study systematically revealed profound changes in the composition and function of the gut microbial community, as well as its interactions with host metabolism, in SD rats 14 days after SCI. We found that SCI induces significant alterations in gut microbiome composition, most notably an increase in *Ligilactobacillus* and a decrease in *Lactobacillus* and *Limosilactobacillus.* These findings are consistent with previous studies, which have reported substantial shifts in gut microbiota following SCI—particularly reductions in anti-inflammatory bacteria such as *Lactobacillus* [15]. Additionally, analyses of species α-diversity and β-diversity further confirmed significant changes in the diversity and structure of the gut microbiome post-SCI. These shifts likely reflect the influence of gastrointestinal environmental changes on microbial community composition after SCI.

Additionally, our species α-diversity index analysis revealed increases in both the Chao1 and ACE indices, suggesting a rise in rare or previously undetected species—findings that contrast with those of Jing et al. [16]. While Jing et al. employed a C57 mouse model and collected samples four weeks post-injury, our study used an SD rat model with sample collection at 14 days post-injury. These methodological differences may account for the discrepancies between the two studies [17]. Although no significant differences were found in the Shannon and Simpson indices, both showed greater stability in the sham group. In contrast, the SCI group exhibited significantly increased within-group variability, potentially indicating reduced gut microbiome stability [18]. Species β-diversity analysis further confirmed substantial changes in gut microbiome composition following SCI, with notable variability among individuals in the SCI group.

Further MaAsLin2 analysis revealed significant differences in key microbial species under SCI conditions, highlighting complex interactions within the gut microbiota that are essential for understanding its role in SCI recovery. A decreased abundance of *Limosilactobacillus* and *Lactococcus*—both producers of beneficial metabolites such as lactate—may impair mucosal health and immune modulation [19, 20]. *Limosilactobacillus* has also been shown to support cognitive functions associated with blood–brain barrier dysplasia and dysfunction [21]. *Romboutsia* is significantly reduced following SCI, and its role appears to be dual. It is a key producer of SCFAs, contributing to gut health and immune regulation [22, 23]. However, under certain stress conditions, its overactivity may promote proinflammatory effects and disrupt host metabolic homeostasis [24].

Conversely, our findings also highlight the detrimental effects of certain pathogens on gut microbiota composition. For instance, *Corynebacterium* and *Macrococcus* were upregulated in the SCI group, potentially contributing to increased neuroinflammation and oxidative stress, which may exacerbate neuronal damage [25–27]. However, some beneficial bacteria, such as *Ligilactobacillus*, were also enriched. *Ligilactobacillus* has been shown to alleviate anxiety- and depression-like behaviors in mice subjected to chronic unpredictable mild stress by modulating tryptophan metabolism. In our study, most pathogens or opportunistic pathogens were present at higher levels in the SCI group, further supporting the hypothesis that gut microbiota may influence recovery following SCI.

Specifically, our analysis of the functional composition of the gut microbiome revealed significant enrichment in key metabolic pathways related to energy metabolism, inflammatory response, and nerve repair, highlighting the potential influence of gut microbiota on SCI recovery [28–31]. Functional β-diversity analysis showed only subtle differences between the two groups, indicating that the overall functional profiles of the microbial communities were largely similar at a macro level. However, MaAsLin2 analysis of eggNOG-enriched pathways identified several differentially abundant pathways, primarily associated with secondary metabolite biosynthesis, carbohydrate metabolism, energy production, lipid metabolism, and amino acid metabolism [32].

The upregulation of secondary metabolite biosynthesis pathways suggests heightened microbial stress responses, potentially involving the production of either anti-inflammatory or proinflammatory compounds [33]. Enrichment of pathways related to carbohydrate and lipid metabolism implies that the carbohydrate intolerance and lipid abnormalities commonly observed after SCI may be linked to gut microbiota dysbiosis [34]. The increased activity in energy metabolism pathways indicates a heightened energy demand by the host, possibly representing a compensatory response to the altered physiological state following SCI [35]. Additionally, alterations in amino acid transport and metabolism point to potential disruptions in neurotransmitter synthesis, immune regulation, and tissue repair processes [36, 37].

In metabolomics analysis, elevated serum metabolites, such as pyruvate and lactic acid, may reflect disordered energy metabolism and local tissue hypoxia in SCI [38]. Additionally, reduced carnosine levels indicate increased oxidative stress, which may exacerbate neuronal damage in SCI [39]. To further investigate metabolic changes, we conducted rno pathway analysis and identified significant alterations in pathways related to energy metabolism, amino acid metabolism, inflammation, and nerve repair. These findings not only provide important insights into the biological basis of SCI but also offer valuable information for developing novel diagnostic markers, assessing injury severity, and guiding treatment strategies. Notably, changes in intestinal microbial community function following SCI were correlated with alterations in serum metabolites. This observation reinforces the strong link between the gut microbiome and host metabolism, particularly in relation to disrupted energy metabolism, elevated oxidative stress, and altered neurotransmitter activity.

Finally, our correlation analysis revealed significant associations between the gut microbiome and serum metabolites, suggesting potential interactions between specific bacterial genera and metabolite profiles. For instance, the positive correlation between *Limosilactobacillus* and carnosine may indicate a role for this genus in alleviating oxidative stress in SCI patients. Additionally, *Limosilactobacillus* appears to influence levels of creatine, pyruvic acid, and lactic acid by degrading nitrite [40–42]. These interactions suggest that the gut microbiome can significantly impact host health and recovery following SCI by modulating the production and regulation of key metabolites [43, 44]. *Limosilactobacillus* is among the most extensively studied strains in clinical research [45]. Notably, *L. reuteri* has shown promise in improving symptoms of hyperactivity and social behavior, as well as in enhancing mental health outcomes related to anxiety and depression in humans [46, 47]. While *Lactococcus* has not yet been widely adopted in oral clinical applications, its probiotic potential is under active investigation. For example, *L. lactis* has been shown to prevent experimental autoimmune encephalomyelitis in mice [48]. Moreover, *L. lactis* can produce GABA through its metabolism, thereby modulating the gut–brain axis [49, 50]. These findings provide new insights into how the gut microbiota can influence CNS disorders. Taken together, our findings underscore the significant impact of the gut microbiome on host metabolism. By altering metabolite levels, the microbiome not only affects immediate biochemical pathways but also plays a pivotal role in overall health outcomes and recovery processes. This growing understanding of the gut microbiota’s metabolic functions opens promising avenues for targeted therapeutic strategies aimed at improving host metabolic health and recovery.

In this study, we combined metagenomics and metabolomics to investigate the effects of SCI on the intestinal microbiome and host metabolism in rats. Our findings offer valuable insights into the potential influence of the gut microbiome on SCI recovery and suggest promising therapeutic strategies for microbiome-based interventions. Below, we summarize the key strengths and limitations of our study: We integrated metagenomic and metabolomic approaches to analyze the impact of SCI on both the gut microbiome and host metabolism, offering a comprehensive view of how microbial and metabolic changes are interconnected in response to injury. Through detailed analyses of gut microbiota composition and host serum metabolites, we observed significant alterations in both microbial structure and metabolic profiles following SCI. These changes point to a potential role of the gut microbiome in modulating host responses related to oxidative stress and energy metabolism, contributing to a deeper understanding of the complex mechanisms underlying SCI. Our findings underscore the therapeutic potential of targeting the gut microbiome as a novel strategy for SCI treatment, laying a scientific foundation for the development of new intervention methods. While we identified associations between changes in the gut microbiome and host metabolism, the specific mechanisms by which these changes influence SCI recovery remain unclear and warrant further investigation. Although our results support the development of gut microbiome-based therapies, their clinical application is still in its early stages. Further research is needed to determine effective intervention methods and evaluate their therapeutic outcomes [51, 52]. Future studies will aim to further validate the relationship between gut microbiota and host metabolism after SCI through fecal microbiota transplantation and supplementation of relevant metabolites.

## Conclusion

In this study, metagenomic and serum metabolomic methods were used to offer a unique perspective and new insights into gut microbiome changes and their impact on host health following SCI. Despite certain limitations, our findings underscore the potential influence of the gut microbiome on SCI recovery, providing a scientific basis for exploring novel therapeutic approaches. However, the specific mechanisms linking the gut microbiome to recovery from SCI require further investigation to assess the effectiveness and safety of potential treatments based on these findings.

## Data Availability

Raw data generated during the current study were deposited in the Zenodo repository. Data can be accessed using the following DOI: 10.5281/zenodo.12696988.
